# How to Regulate the Migration Ability of Emulsions in Micro-Scale Pores: Droplet Size or Membrane Strength?

**DOI:** 10.3390/molecules28041672

**Published:** 2023-02-09

**Authors:** Qi Sun, Zhao-Hui Zhou, Lu Han, Xin-Yuan Zou, Guo-Qiao Li, Qun Zhang, Fan Zhang, Lei Zhang, Lu Zhang

**Affiliations:** 1 Key Laboratory of Photochemical Conversion and Optoelectronic Materials, Technical Institute of Physics and Chemistry, Chinese Academy of Sciences, Beijing 100190, China; 2 University of Chinese Academy of Sciences, Beijing 100049, China; 3 State Key Laboratory of Enhanced Oil Recovery, PetroChina Research Institute of Petroleum Exploration & Development, Beijing 100083, China; 4 No.2 Oil Production Company, Daqing Oilfield Company Limited, Daqing 163000, China

**Keywords:** surfactant, interfacial tension, interfacial membrane strength, emulsion viscosity, microscopic visualization, oil displacement efficiency

## Abstract

Micro visualization has become an important means of solving colloid and interface scientific problems in enhanced oil recovery. It can establish a relationship between a series of performance evaluations of an oil-water interface under macroscopic dimensions and the actual application effect in confined space, and more truly and reliably reflect the starting and migration behavior of crude oil or emulsion in rock pores. In this article, zwitterionic surfactant alkyl sulfobetaine (ASB) and anionic extended surfactant alkyl polyoxypropylene sulfate (A145) were employed as flooding surfactants. The macroscopic properties of the surfactant solutions, such as the oil-water interfacial tension (IFT), the interfacial dilational rheology and the viscosity of crude oil emulsions, have been measured. At the same time, we link these parameters with the oil displacement effect in several visual glass models and confirm the main factors affecting the migration ability of emulsions in micro-scale pores. The experimental results show that ASB reduces the IFT through mixed adsorption with crude oil fractions. The flat arrangement of the large hydrophilic group of ASB molecules enhances the interactions between the surfactant molecules on the oil-water interface. Compared with sulfate, betaine has higher interfacial membrane strength and emulsion viscosity. A145 has a strong ability to reduce the IFT against crude oil because of the larger size effect of the PO chains at the oil side of the interface. However, the membrane strength of A145 is moderate and the emulsion does not show a viscosity-increasing effect. During the displacement process, the deformation ability of the front emulsions or oil banks is the main controlling factor of the displacement efficiency, which is determined by the membrane strength and emulsion viscosity. The strong interfacial membrane strength and the high emulsion viscosity are not conducive to the migration of droplets in pore throats and may result in low displacement efficiency.

## 1. Introduction

With the advancement of tertiary oil recovery technology in recent years, the excellent properties of surfactants in improving oil recovery have been fully recognized. The chemical flooding method, in which surfactants are added to the flooding fluid, is considered to be one of the most effective means to improve oil recovery [1,2,3,4]. Research on the structure-activity relationship of surfactants used in oil displacement has aroused the continuous interest of scholars at home and abroad, particularly the optimization of the oil displacement system in high temperature, high salt and low permeability reservoirs [5,6,7]. Compared with conventional oil reservoirs, low permeability restricts the efficient use of polymers for oil displacement. On the other hand, high temperature and high salt oil reservoirs have higher requirements for the temperature and salt resistance of surfactants; ordinary surfactants, such as anionic petroleum sulfonate, non-ionic Triton TX-100, etc., will no longer be able to meet the application needs. Zwitterionic surfactants and anionic nonionic surfactants have gradually become the most suitable choice.

Zwitterionic surfactants have the characteristics of strong interfacial activity, low toxicity, and good biodegradability [8]. As a typical zwitterionic surfactant for oil displacement, betaine has both a negative and positive center in its hydrophilic group. It exists in a neutral form in a wide pH range and has strong adaptability to high salinity [9]. There have been many studies on the properties of betaine, such as reducing its oil-water interfacial tension (IFT), increasing its oil-water interfacial membrane strength, modifying its solid surface wettability, etc. [10,11,12,13].

Anionic nonionic surfactants, also called extended surfactants, refer to a new type of surfactant with a weak oil-soluble polyoxypropylene (PPO) chain or water-soluble polyoxyethylene (PEO) chain between the hydrophobic alkyl group and the hydrophilic head group of traditional surfactants [14]. In recent years, there has also been a lot of research on the role of extended surfactants in enhancing oil recovery. Kang [15] synthesized a class of carboxylate-based extended surfactants (n-C_12-14_EPC) and characterized its good electrolyte tolerance. Ge [16] studied the IFT of sulfate-based extended surfactant (NPP_3_S) and different weak base compound systems, and found that it has a good synergistic effect with Na_2_CO_3_ in reducing the oil-water IFT. Jiang [17] explored the wettability of anionic nonionic surfactants on polymethyl methacrylate and investigated the influence of alkyl branching on the adsorption behavior.

The traditional laboratory evaluation of the surfactant used in oil recovery focuses on the reduction in the oil-water IFT, with the belief that an ultra-low IFT is a necessary condition for improving oil recovery. In addition, most of the evaluation methods are carried out under macroscopic conditions, which is inconsistent with actual rock pores. However, for core flooding experiments [18,19,20], although it can simulate the real reservoir environment to the greatest extent, important information, such as the migration behavior of oil-water mixtures in rock pores during the displacement process and the occurrence state of the remaining oil in the pores after displacement is complete, cannot be visually reflected, which provides limited reference for the exploration of enhanced oil recovery.

In recent years, micro-models have become popular experimental tools for two-phase flow studies [21,22], there have been many reports on the size design, material selection, manufacturing method and application details of the micro-flow model [23], and the emergence of micro-visualization methods has made particularly prominent contributions to the evaluation of oil recovery efficiency [24,25,26,27]. Xie [28] focused on the influence of the non-Newtonian fluid phase viscosity on the oil displacement efficiency in homogeneous and heterogeneous models, and found that the shear thickening had a better displacement effect on the crude oil in the heterogeneous model, while in the homogeneous model, the difference between the shear thickening and shear thinning is not obvious. Lei [29] used the Changqing sandstone reservoir in China as the research background and employed the method of microfluidic experiments to investigate the superiority of micro-gel particle suspensions (MGPS) compared to conventional hydrolyzed polyacrylamides (HPAM) in a circular heterogeneous structure in improving oil recovery. He believes that the process of MGPS flowing in porous media will cause the automatic redistribution of the particle concentration, which in turn leads to strong fluid field fluctuations in the medium and has a better effect of recovering crude oil than the increase in the bulk viscosity.

Even though the research on the visual oil displacement efficiency of surfactants has already begun, most of the investigations focus on ionic surfactants [30,31,32]. Explorations of zwitterionic surfactants and anionic nonionic surfactants with a relatively larger molecular size and more complex interfacial behavior are rare. The distribution of pore throats in the microfluidic model is mostly homogeneous, and the investigation of the flow characteristics of crude oil emulsions in various heterogeneous pores needs to be perfected [33,34]. Furthermore, the existing visual flooding experiments mainly investigate the capillary force, capillary backpressure, Haines jump, flow field changes and other physical parameters or phenomena in the process of theoretical analysis [35,36]. Less attention has been paid to the physical chemical behavior of surfactant molecules on the oil/water/solid interface.

In general, the currently available commercial, cheap surfactants are mainly ionic, which are not suitable for special reservoirs with high temperature and high salt. With the development of oilfield exploitation, conventional surfactants cannot meet the needs of harsh reservoir conditions. In this article, two types of emerging surfactants, betaine ASB and extended surfactant A145, which have been gradually recognized and adopted in high temperature and high salinity oilfields, will be fully explored and compared in terms of their respective abilities in reducing the oil-water IFT, improving the interfacial membrane strength, changing the emulsion viscosity, regulating wettability and so on. Furthermore, their displacement effects on crude oil in visual glass models, including parallel straight channels, heterogeneous fractures and simulation homogeneous models, have been tested. Finally, the micro-dynamic behaviors of the emulsion droplets in the models, such as snapping or deformation through the pore throat, were connected with a series of interface properties under macroscopic conditions, which enabled us to identify the main control factors that affect the migration ability of droplets in enhanced oil recovery.

## 2. Results and Discussion

### 2.1. Oil-Water IFT

**Figure 1:**
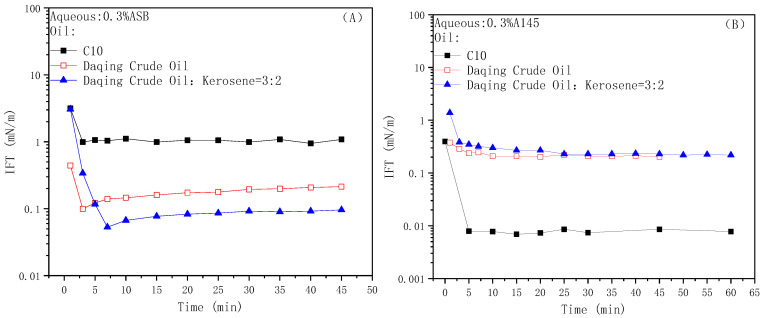
Dynamic IFTs of ASB (**A**) and A145 (**B**) solutions against n-decane, Daqing crude oil and Daqing diluted oil.

The dynamic IFTs of the zwitterionic surfactant betaine ASB against n-decane, Daqing crude oil and Daqing crude oil diluted with kerosene are shown in Figure 1A. Among them, the test temperature with the diluted crude oil is 25 °C, the others are 45 °C. Compared with the Daqing crude oil and diluted crude oil, the IFT between the ASB solution and n-decane is obviously higher because the hydrophilic group size of the ASB molecule is much bigger than the hydrophobic group. In the previous work, we calculated the saturated adsorption area of ASB using the Gibbs adsorption formula and detected the arrangement of the ASB molecules at the interface through molecular dynamics simulation. It is proven that the whole hydrophilic group of betaines is tiled on the interface and determines the adsorption area. The space occupied by the ionic head group at the water phase side is much larger than the long linear alkyl chain at the oil phase side, and the spatial matching cannot be satisfied [37]. When active components are present in the oil phase, the IFT is significantly reduced, which is consistent with our previous work [9,38]. Active fractions in the crude oil will mix and be adsorbed with the betaine molecules from the oil phase side to form a compact adsorption membrane, resulting in a synergistic effect with the surfactant.

Figure 1B shows the dynamic IFTs of anionic nonionic surfactant A145 against n-decane, Daqing crude oil and Daqing crude oil diluted with kerosene. In contrast to ASB, the interfacial tension value between A145 and C_10_ is very low, indicating that A145 itself has a good ability to reduce the IFT. Similarly, we have simulated the configuration and arrangement of the PO chain segment of the extended surfactant through parameters such as the radius of gyration and the inclination angle. It was found that as the adsorption amount gradually increased, the entire PO chain segment spiraled upright in the oil phase in a “thin cylinder” manner, and the occupied area was approximately equal to the molecular area. In other words, the oxypropylene group (PO) in the A145 molecule has an ability to “stack horizontally and stretch vertically” at the oil phase side, which can achieve a good spatial matching effect with the hydrophilic side [39,40]. The IFTs of the A145 solution against Daqing crude and diluted oil are maintained at the order of 0.1 mN/m, proving that the active components in the crude oil have competitive adsorption with the A145 molecules on the interface, destroy the tight adsorption film when the A145 molecule exists alone, and result in an anti-synergistic effect [41].

### 2.2. Oil-Water Interface Membrane Strength

**Figure 2:**
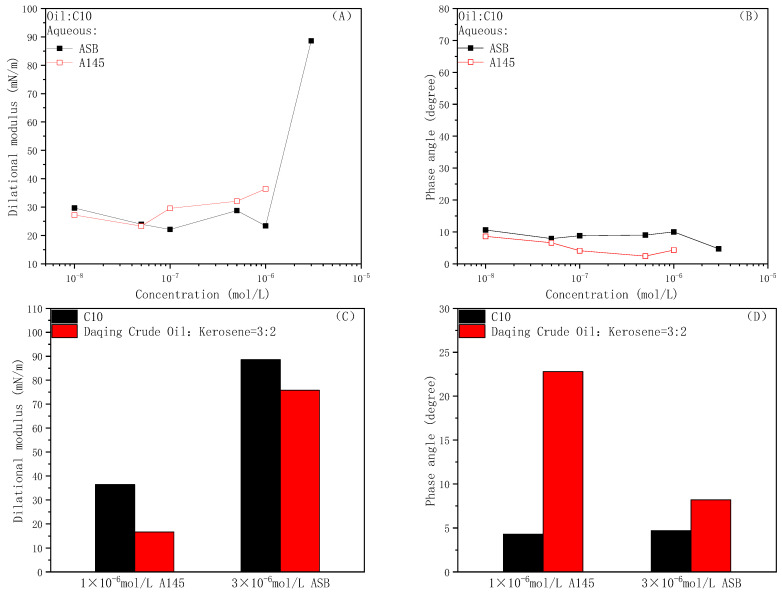
Interface dilational modulus (**A**) and phase angle (**B**) of ASB and A145 solutions against n-decane and diluted oil as a function of surfactants concentration, comparison of the dilational modulus (**C**) and phase angle (**D**) of surfactants with n-decane and diluted crude oil at the same concentration.

The IFT is a direct reflection of the adsorption amount for the surfactant molecules on the oil-water interface. However, in order to obtain some information related to the interface membrane strength, such as the interactions between the surfactant molecules on the oil-water interface and the diffusion-exchange rate of the surfactant molecules between the interface and bulk, we must turn to the means of interfacial dilational rheology. The interfacial dilation rheological results of the two surfactant solutions are shown in Figure 2. Dot-line in graph (A) is the curve for the interfacial dilational modulus as a function of the concentration when the oil phase is n-decane, and histogram (C) is the numerical comparison of the modulus against the different oil phases at the same concentration. Similarly, the dot-line exhibits the curve of the dilational phase angle as a function of the concentration when in n-decane (B), histogram (D) is the comparison of the phase angle against the different oil phases at the same concentration.

In the process of the periodic expansion and compression of the interface area, the interface membrane formed by the betaine molecules resists deformation by changing the orientation of the hydrophilic group; rather than the diffusion exchange between the bulk phase and the interface, the rearrangement process inside the membrane is dominant as a result [42]. Therefore, the interface membrane stores large energy when subjected to deformation disturbances, the interfacial dilational modulus can reach about 80 mN/m before the cmc (about 1.6 × 10^−6^ mol/L), whether the oil phase is C_10_ or Daqing diluted oil, which is much greater than that of conventional ionic surfactants. Moreover, the dilational phase angle is always small (lower than 10 degree). In a word, the betaine adsorption membrane has high strength and elasticity.

For anionic nonionic surfactant A145, similarly to the surfactants with ethylene oxide at the hydrophilic group side [43,44,45], the interfacial dilational modulus of A145 reaches a plateau value near 35 mN/m. As the concentration increases, the PO groups of the surfactant molecules will spontaneously stack and spiral up in the oil, forming a spatial configuration similar to a thin cylinder as a whole, instead of random stacking and winding along the interface laterally [39]. In addition, the overall chain link is relatively soft and the interaction of PO-containing A145 molecules on the oil-water interface is very weak; at this moment, the diffusion exchange determines the membrane properties. It is worth pointing out that the modulus against the diluted crude oil is lower than that against n-decane and the phase angle is always larger, indicating that the presence of active fractions in the crude oil will destroy or mask the interactions between the surfactant molecules on the oil-water interface. In conclusion, the A145 adsorption layer is an elastic membrane with moderate strength.

### 2.3. Viscosity of Crude Oil Emulsion

**Figure 3:**
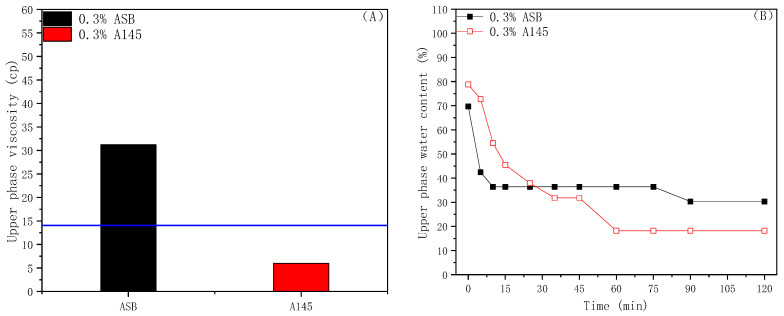
Upper phase viscosity (**A**) and water content (**B**) of ASB and A145 crude oil emulsion.

The upper phase viscosity and water content of the emulsion measured at a constant temperature of 45 °C after two hours are shown in Figure 3; the blue line in (A) is the initial viscosity of the crude oil at the reservoir temperature. Betaine ASB has a better viscosity-increasing effect and a higher upper phase water content than A145, as shown in (B), which may have contributed to the higher strength of the oil-water interface membrane formed by betaine molecules. Therefore, because of the synergistic effect of the crude oil fractions for bulk thickening, the dispersed water droplets with the high strength membrane in the crude oil emulsion show an effect of improving the viscosity, even at high water-containing conditions. On the contrary, the viscosity of the upper W/O emulsion formed by the A145 solution is obviously lower than the initial value, indicating the effect of the dilution on the crude oil by the water droplets.

### 2.4. Three Phase Contact Angle

**Figure 4:**
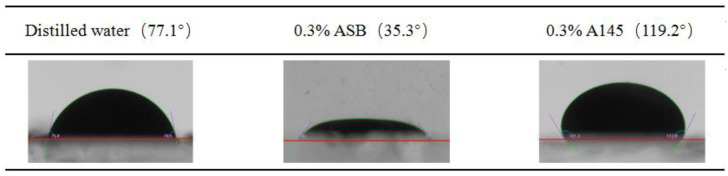
Macro-static crude oil/water/solid three-phase contact angles.

The experimental results of the macro-static crude oil/water/solid three-phase contact angles are shown in Figure 4. The peeling ability of the two surfactants to the oil membrane on the quartz surface is completely different. ASB molecules can hydrophobically modify the quartz surface through Lifshitz–van der Waals interactions [46]. As a result, the solid-liquid IFT is greater than the sum of the oil-water IFT component along the direction of the quartz surface and the oil-solid interfacial tension, on the position of the three-phase contact point, and the oil droplets stick to quartz surface in a manner similar to a “coin”. A145 molecules weakly adsorb on the quartz surface through polar action [47]; electrostatic repulsion between the oil-water interface and solid surface will produce a separation pressure at the three-phase contact point and greatly enhance the ability to peel off the oil membrane. The oil droplet protrudes from the quartz surface and the three-phase contact angle value becomes much larger.

### 2.5. Evaluation of Oil Displacement Effect in Visual Glass Model

**Figure 5:**
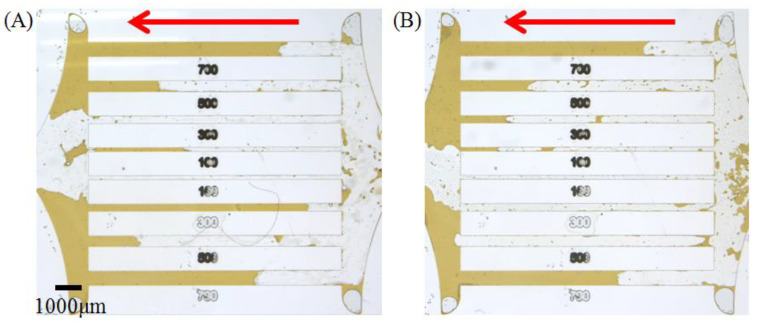
The final state of crude oil displacement of ASB (**A**) and A145 (**B**) solutions in parallel straight channel model.

Combining the above investigations of physical chemical parameters such as IFT, interfacial dilational rheology, crude oil emulsion viscosity and three-phase contact angle, the displacement effect of the two surfactant solutions on crude oil in different glass models were tested, respectively. Figure 5 shows the remaining state of the crude oil in the parallel straight channel model after the experiment, where the red arrow indicates the inject direction of the surfactants solution.

Both types of surfactants have a better sweeping effect in the straight channel model. However, A145 can completely break through more straight channels than ASB. This is reasonable because the higher membrane strength and emulsion viscosity restrict the activation of the oil bank at the front end of channel. ASB can reduce the IFT of diluted crude oil to 0.09 mN/m, and originally had an advantage in starting crude oil in contrast with 0.2 mN/m of A145. However, the frictional resistance brought by the viscosity at this time obviously limits the fluid migration in the straight channel, and occupied a dominant position in the factors affecting the oil displacement effects [48].

**Figure 6:**
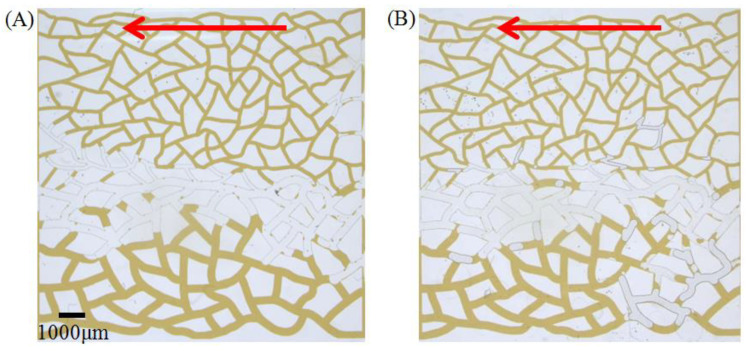
The final state of crude oil displacement of ASB (**A**) and A145 (**B**) solutions in heterogeneous fracture model.

The heterogeneous fracture model is more similar to the rock pores distribution in low-permeability reservoirs. Here, we examined the effect of two types of surfactants during crude oil displacement in a heterogeneous model and the remaining oil situations after the experiment are shown in Figure 6. Comparing the displacement area and the position of these two surfactants in the model, one can see that the displacement area of the A145 solution almost concentrated on the large pore side with high permeability, while the ASB solution is distributed on both the high permeability and low permeability parts. During the migration of the crude oil emulsion, the resistance generated by the high-viscosity of the front oil wall continues to increase, and the solution starts to move along the large and small pores at the same time. This demonstrated that a certain crude oil emulsion viscosity is beneficial to improve the sweep efficiency of surfactant solutions in low-permeability oil reservoirs.

**Figure 7:**
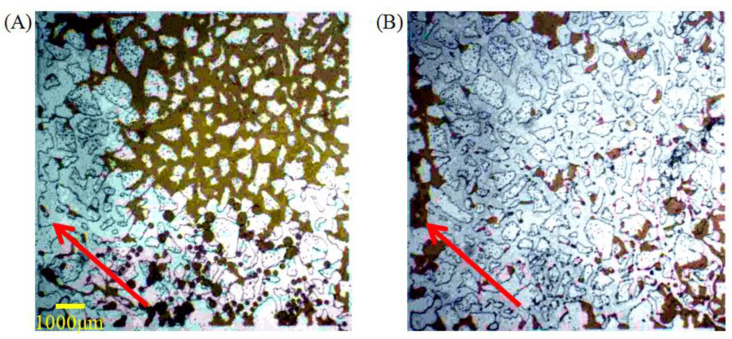
The final state of crude oil displacement of ASB (**A**) and A145 (**B**) solutions in homogeneous simulation model.

The oil displacement effects of the two surfactant solutions in the simulated homogeneous model with a larger pore throat volume and higher pore connectivity are shown in Figure 7A,B, respectively. Connecting with the research ahead, the oil displacement efficiency of the ASB and A145 solutions in the three visual glass models for diluted crude oil are statistically compared and the results are shown in Table 1. Without exception, the extended surfactant A145 with lower oil-water interfacial membrane strength shows the best oil displacement in all of the types of channels, which seemed to predict a “positive correlation” between low membrane strength and high oil recovery, not low IFT.

### 2.6. Remaining Oil Type Analysis in Visual Glass Model

**Figure 8:**
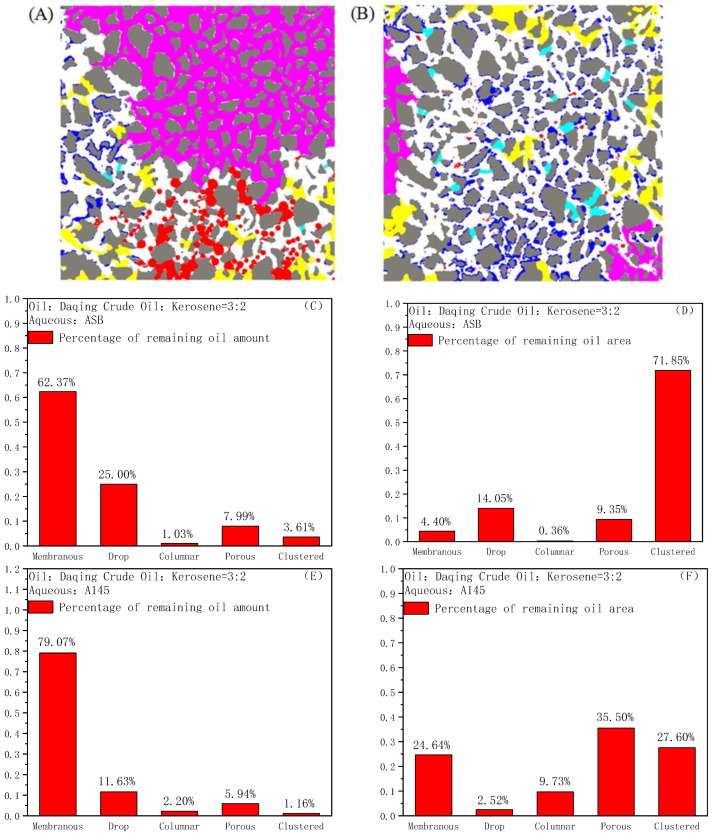
The remaining oils after ASB (**A**) and A1045 (**B**) flooding in simulated homogeneous model; The amount (**C**) and area (**D**) distributions of remaining oil types after ASB flooding; The amount (**E**) and area (**F**) distributions of remaining oil types after A145 flooding.

The remaining oil type analysis software can be used to classify the remaining crude oil in the models. The remaining oil, the thickness of which was less than 1/3 of the pore throat diameter, is defined as a membrane, whereas a size less than or equal to the diameter of the pore throat, but not in contact with the throat, is defined as a droplet, otherwise defined as a columnar. In addition, a number of connected pores in the throat greater than five are defined as clusters, otherwise as porous. We have given them blue, red, cyan, rose and yellow color to the membrane, droplet, columnar, cluster and porous, respectively [49]. By the above classification, the quantity and area statistics distribution percentage of the different types of remaining oil are obtained and shown in Figure 8.

For ASB, after the displacement, the remaining oil in the model is dominated by clusters, with an area distribution reaching 71.85% of the total remaining oil, and a large amount of crude oil in the pores has not been activated, as shown in Figure 8A. In addition, the total area of the droplet and porous among the remaining oil also reaches 23.4%, indicating that even if some crude oil was activated, it is difficult to migrate through the pore throats. In short, the displacement efficiency in the simulation homogeneous model is very low. On the other hand, for A145, the remaining oil types, after flooding, are completely different from those of ASB, as shown in Figure 8B. The remaining oils are mainly composed of droplets, membranes and pores. Among them, the number of membranes accounted for 79.07% of the total remaining oil, indicating that almost all of the crude oil in the model is started and affected, and the replaced efficiency is much higher.

**Figure 9:**
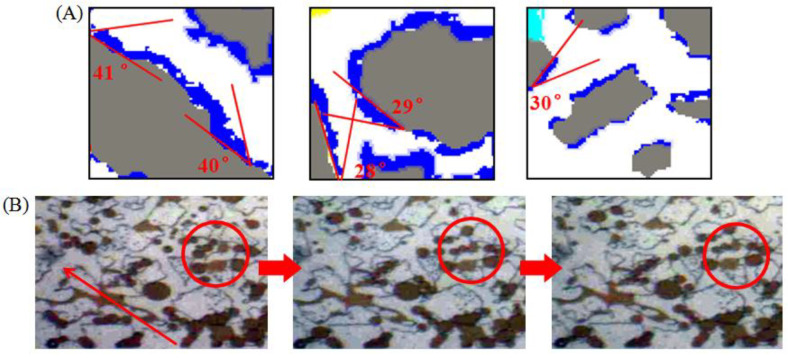

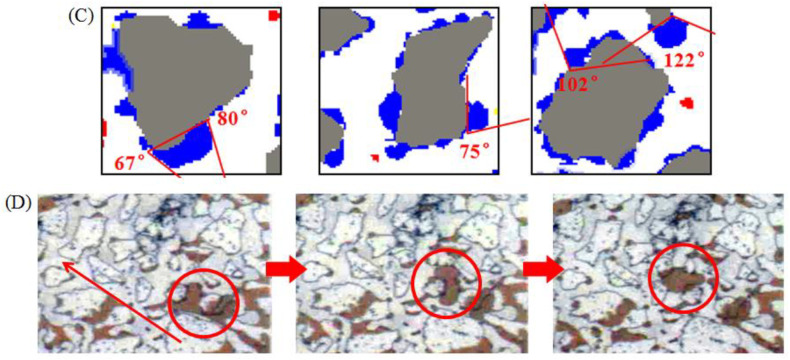
The microscopic three phase contact angles of membranous remaining oil in simulated homogeneous model after ASB (**A**) and A145 (**C**) flooding; The movement of crude oil emulsion in pore throat during ASB (**B**) and A145 (**D**) displacement.

Moreover, the measurements of the oil/water/solid three phase contact angle of the membranes in the homogeneous simulation model were also carried out. Having taken the contact angle on the side of oil phase, some of the measured values of ASB are shown in Figure 9A, and the measurement error is less than ±1.5°. The contact angle is distributed below 40° and oil droplets have a tendency to spread on the surface of the tunnel, which is consistent with the value obtained from the macroscopic static three-phase contact angle test. For A145 in Figure 9C, the contact angle was distributed between 60° and 120°, also corresponding to the macro-static measurement. Furthermore, in terms of the difficulty to restart the remaining oil in the reservoirs after water flooding, a larger oil/water/solid three-phase contact angle is also more advantageous.

Concurrently, to deeply understand the initiation and migration mechanism of these two types of surfactant solutions for crude oil, the movement of the crude oil emulsion in the simulated homogenization during the displacement of ASB with high oil-water interface membrane strength is clearly presented Figure 9B. Only part of the smaller oil droplets can move continuously through the throat until it is replaced completely, and the amount of large-size oil droplets or oil walls were trapped in pore throat without any deformation ability under the driving force. On the contrary, as shown in Figure 9D, the crude oil droplets, with a small size or a size similar to the width of the throat, can pass through any pore throat for A145, and even the characteristics of “migration after drawing” can appeared under the promotion of a low oil-water IFT. Moreover, the larger oil droplets or oil walls can also change their shape at will under the action of the driving force and, finally, pass through the pore throat. This good deformability is attributed to its low oil-water IFT and interface membrane strength. Comparing the migration characteristics of the ASB emulsions with the same pore throat size, we can reach the conclusion easily that it is the deformation ability of the oil droplets, which is determined mainly by the strength of the oil-water interface membrane, not the droplet size, that acts as the crucial factor to control the oil droplets passing through the pore throat or not.

## 3. Experiment Section

### 3.1. Materials

**Figure 10:**
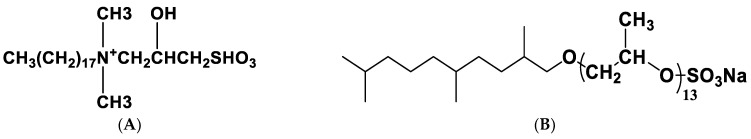
Structural formula of ASB (**A**) and A145 (**B**).

The zwitterionic surfactant alkyl sulfobetaine (ASB) was synthesized in our lab with a purity above 95 mol%, which was checked by elemental analysis and ^1^H nuclear magnetic resonance (NMR) spectroscopy [9], from Beijing, China. The anionic extended surfactant, alkyl polyoxypropylene sulfate (A145), was purchased from Sasol (China) Chemical Co., Ltd. (Shanghai, China) [50]. The structures of these two surfactants are shown in Figure 10A,B. The nmin values of ASB and A145, obtained by the n-alkane interfacial tension scanning, which can evaluate hydrophilic-lipophilic balance of surfactants, are 5 and 10, respectively. The larger the nmin value, the stronger the oil solubility of the surfactant. Therefore, ASB is a water-soluble surfactant, while A145 has a certain solubility in both oil and water phases.

Double distilled water (resistivity > 18.2 MΩ·cm) was used in the preparation of the surfactant solutions, with an approximate pH value of 7.0. At the same time, sodium chloride with a mass concentration of 0.5% was added to meet the salinity requirements. The crude oil in this paper came from the Daqing Oilfield of China and the crude oil fractions and acid value are shown in Table 2, respectively [51]. The kerosene used in the research was purified by silica gel column chromatography (100–200 mesh), and its IFT against pure water reached a stable value of 40 mN/m. The n-decane was purchased from the Macklin reagent company and the purity reached analytical purity. In this paper, we take a block in Daqing Oilfield as the research background; unless specifically stated, the temperature is fixed at 45 °C (reservoir temperature) and the concentration of surfactant is fixed at 0.3% (field concentration) in all experiments.

**Figure 11:**
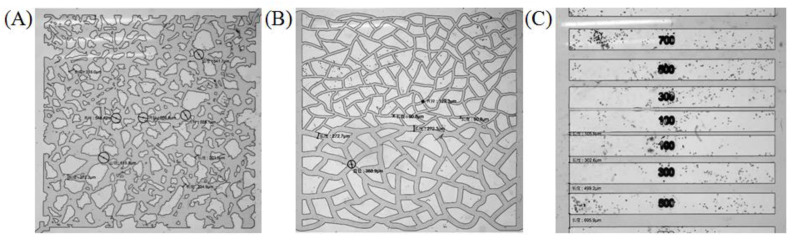
Simulation homogeneous model (**A**), heterogeneous fracture model (**B**) and parallel straight channel model (**C**). Black painting in the images is the corresponding dimensioning.

The glass models of the microscopic visualization experiment came from China University of Petroleum (Beijing), State Key Laboratory of Oil and Gas Resources and Exploration. Among them, the diameter of the hole in the simulation homogeneous model is 500~600 μm and the width of the throat is 200~300 μm. The throat widths in the parallel straight channel model are 700, 500, 300 and 100 μm, respectively. In the heterogeneous fracture model, the diameter of the pores in the high permeability part is about 390 μm and the throat width is about 270 μm, while the diameter of the pores in the low permeability part is about 130 μm and the throat width is about 90 μm. The specific situations are shown in Figure 11.

### 3.2. Visualized Crude Oil Displacement Measurement

The experiment device of the visualized crude oil displacement test was provided by Beijing Eastern-Dataphy Instrument Co., Ltd., from Beijing, China. Before the experiment, Daqing crude oil and kerosene were formulated into diluted crude oil at a mass ratio of 3:2, and the viscosity at room temperature (25 °C) was 22 cP. Then, the diluted crude oil was injected into different types of glass models at an injection rate of 5 mL/min until the oil filled the entire glass model and there were no bubbles in the pore throats.

During the experiments, different types of surfactant solutions were injected into the glass model at a constant injection rate of 0.1 μL/min, until the amount of crude oil in the model no longer changed. The entire process of the displacement of the diluted crude oil in the glass models was recorded through the video mode of the microscope. It should be pointed out that the displacement direction of the simulated homogeneous model in this paper is from the lower right to the upper left of the parallel straight channel model and the heterogeneous fracture model are both from right to left.

Finally, the remaining oil type and the displacement efficiency are analyzed after the displacement process. Moreover, the activation, migration and matching mechanism of the different types of crude oil emulsions in different sizes and types of pore throats are obtained. The measurement error is less than 5%.

### 3.3. Oil-Water IFT Measurement 

The IFT data obtained in this paper are all measured using the spinning drop method with Texas-500c interface tension meter from Beijing Shengwei Technology Co., Ltd. (Beijing, China). During the measurements, the rotational speed was input at 5000 r/min. In all cases, the standard deviation did not exceed ±5% when the IFT was lower than 0.1 mN/m [9].

### 3.4. Interfacial Dilational Rheology Measurement

The evaluation of the oil-water interface membrane strength in this article, that is, the test of the interface dilational rheology, was completed by the LSA100OEDM optical dilational rheometer, from Beijing Eastern-Dataphy Instrument Co., Ltd. (Beijing, China). Using a curved needle, an 8 μL oil droplet (N-decane or Dilute crude oil) was squeezed into the surfactant solution and apply a sinusoidal disturbance with a frequency of 0.1 Hz and an amplitude of 1.5 μL to the oil droplet through an oscillator. At the same time, a high-speed camera is exploited to capture the profile of the oil drop in the surfactant solution and transmit it to the data acquisition computer in real time. The profile is fitted by the Laplace equation; the dynamic value of the IFT with the interface area changed will be obtained. Until the IFT value no longer changed within 30 min, the test is considered complete.

As we described before, when the interface is periodically compressed and expanded, the IFT also changes periodically. The dilational modulus is defined as the ratio of the change on the IFT to the relative change on the interface area, which is [52,53,54]:

(1) $$\varepsilon =\frac{d\gamma }{dlnA}$$

where *ε* is the dilational modulus, *γ* is the IFT, *A* is the interfacial area.

For a viscoelastic interface, there is a certain phase difference *θ* between the periodic change of the IFT and the periodic change of the interface area, which is called the phase angle of the dilational modulus. The measurement errors are ±1 mN/m and ±2 degree for the modulus and phase angle, respectively.

### 3.5. Crude Oil Emulsion Viscosity Measurement

Daqing crude oil and two surfactant solutions are fully emulsified by hand shaking for 100 times at a mass ratio of 5:5. The obtained crude oil emulsion was left to stand in an incubator, the demulsification and water separation at different times was photographed and recorded. The concept of the water content in the upper phase is introduced here; that is, the ratio of the water volume in the emulsion to the total volume of the emulsion. When the stability of the emulsion no longer changes, a 1 ml sample was taken from the upper phase for viscosity measurement through Brookfield DV3T viscometer, the rotor model is 42 CP, rotational speed is fixed at 6.0 RPM. The measurement error is lower than 1%.

### 3.6. Oil/Water/Solid Three Phase Contact Angle Measurement

The quartz pieces were ultrasonicated with acetone, ethanol, and ultrapure water for 30 min in turn [46]. After drying with a hairdryer and standing for 1 h, 2 μL Daqing crude oil was dropped onto the quartz surface and placed in a constant temperature box for aging for about 10 min. Next, it was immersed upside down in a quartz tank containing surfactant solution with the oil drop facing down [55]; the temperature was maintained at 45 °C by heating in a water bath. LSA100OEDM was from Beijing Eastern-Dataphy Instrument Co., Ltd. (Beijing, China) and was used to measure the three-phase contact angle, and the measurement error is less than ±1.5°. The contact angle reached the steady state value after 10 min.

## 4. Conclusions

**Figure 12:**
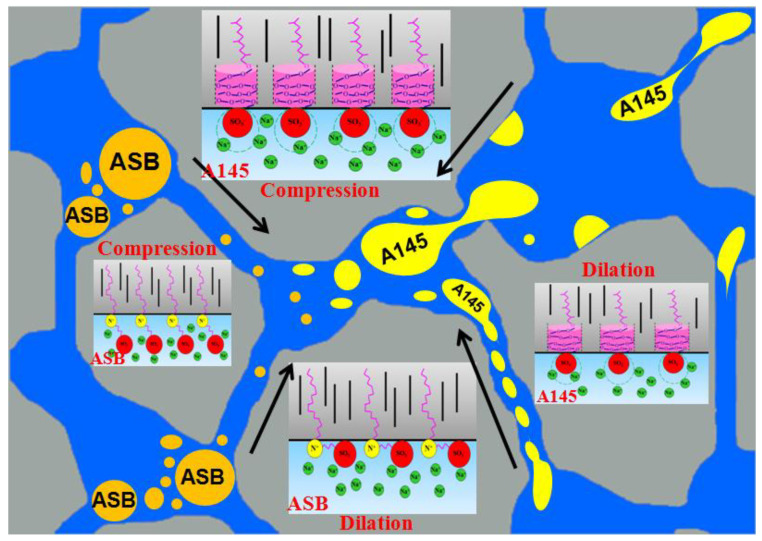
Schematic diagram of the arrangement of surfactant molecules on oil-water interface before and after the droplets pass through pore throat.

In this paper, taking the measurements of physical chemical parameters, such as the oil-water IFT, interfacial dilational rheology, and crude oil emulsion viscosity at the macro-scale, as the basis, and by employing the microscopic visualization flooding experiments as a characterization method, we linked the oil-water interface behavior of different types of surfactants under macroscopic conditions with the start-up and migration capabilities of crude oil emulsions at micro scale. In addition, combined with the previous molecular simulation work and the experimental data in this paper, the possible mechanisms that affect the efficiency of oil movement in pores have been obtained, as shown in Figure 12, and conclusions are as follows:

(1) Zwitterionic surfactant ASB can generate a synergistic effect with the active fractions in crude oil and greatly reduce the oil-water IFT. Moreover, the flat arrangement of the large hydrophilic group of ASB molecules enhances the interactions between the surfactant molecules on the oil-water interface. Therefore, the dilational modulus is much greater than that of conventional surfactants. As a result, the ASB membrane has high strength in nature and the viscosity of the crude oil emulsion increases significantly.

(2) Anionic extended surfactant A145 itself has a strong ability to reduce the IFT against crude oil, which is due to the larger size effect of the PO chains at the oil side of the interface. However, the helical extension of the PO group in the direction perpendicular to the oil-water interface does not result in an enhancement of the interactions between the surfactant molecules. The membrane strength of A145 is moderate and the emulsion does not show a viscosity-increasing effect.

(3) Lower oil-water IFT is necessary for the start of crude oil and favorable for the migration in pores. However, compared with factors such as the IFT, droplet size and membrane strength, the deformability of the droplet, which is determined mainly by the strength of the oil-water interface membrane, is the crucial factor to control the migration of crude oil emulsion in the pores and affects the displacement efficiency. That is to say, in this case, the extended surfactant A145 is more suitable for oil recovery from rock than zwitterionic surfactant ASB.

## Figures and Tables

**Table 1:** The oil displacement efficiency of ASB and A145 solutions in three visual glass models.

	Glass Model	Parallel Straight Channel	Heterogeneous Fracture	Homogeneous Simulation
Surfactant				
ASB		53.6%	21.6%	49.7%
A145		66.0%	24.7%	86.3%

**Table 2:** The properties of Daqing crude oil.

Oil	Acid Value (mgKOH/g)	Saturate %	Aromatic %	Resin %	Asphaltene %
Daqing crude oil	0.12	73.11	8.32	18.41	0.16

## Sample Availability

Samples of the compounds are not available from authors.
